# Six-year mortality associated with living alone and loneliness in Swedish men and women born in 1930

**DOI:** 10.1186/s12877-023-04503-y

**Published:** 2023-12-01

**Authors:** Masuma Novak, Margda Waern, Lena Johansson, Anna Zettergren, Lina Ryden, Hanna Wetterberg, Therese Rydberg Sterner, Madeleine Mellqvist Fässberg, Pia Gudmundsson, Ingmar Skoog

**Affiliations:** Institute of Neuroscience and Physiology, Department of Psychiatry and Neurochemistry, Centre for Ageing and Health (AgeCap), Sahlgrenska Academy, the University of Gothenburg, Gothenburg, Sweden

**Keywords:** Living alone, Loneliness, Mortality, Gender, Sweden, Epidemiology

## Abstract

**Background:**

This study examined how living alone and loneliness associate with all-cause mortality in older men and women.

**Methods:**

Baseline data from the Gothenburg H70 Birth Cohort Studies, including 70-year-olds interviewed in 2000 and 75-year-olds (new recruits) interviewed in 2005 were used for analyses (*N* = 778, 353 men, 425 women). Six-year mortality was based on national register data.

**Results:**

At baseline, 36.6% lived alone and 31.9% reported feelings of loneliness. A total of 72 (9.3%) participants died during the 6-year follow-up period. Cumulative mortality rates per 1000 person-years were 23.9 for men and 9.6 for women. Mortality was increased more than twofold among men who lived alone compared to men living with someone (HR 2.40, 95% CI 1.34–4.30). Elevated risk remained after multivariable adjustment including loneliness and depression (HR 2.56, 95% CI 1.27–5.16). Stratification revealed that mortality risk in the group of men who lived alone and felt lonely was twice that of their peers who lived with someone and did not experience loneliness (HR 2.52, 95% CI 1.26–5.05). In women, a more than fourfold increased risk of mortality was observed in those who experienced loneliness despite living with others (HR 4.52, 95% CI 1.43–14.23).

**Conclusions:**

Living alone was an independent risk factor for death in men but not in women. Mortality was doubled in men who lived alone and felt lonely. In contrast, mortality was particularly elevated in women who felt lonely despite living with others. In the multivariable adjusted models these associations were attenuated and were no longer significant after adjusting for mainly depression in men and physical inactivity in women. Gender needs to be taken into account when considering the health consequences of living situation and loneliness.

## Introduction

Sweden has the highest proportion of older adults living alone worldwide [1], with approximately 31% of men and 59% of women aged 75 and older currently living alone [2]. The high prevalence of solo-living in Sweden is primarily due to long-life expectancies, decrease in family size, decrease in intergenerational co-residence, and ageing-in-place welfare policies that facilitate the difficult aspects of living alone in the home [3].

Living alone and loneliness are distinct concepts: loneliness is commonly defined as subjective negative feelings about one’s level of social contacts than desired [4], whereas living alone is an objective measure of one’s living arrangement. Living alone, particularly in the western societies, may be a choice (desirable) or an undesirable situation, while loneliness always reflects an undesirable situation. Individuals who live alone are not necessarily lonely and vice versa. Previous research has shown a harmful effect of loneliness on all-cause mortality [5, 6], and the effect seems slightly stronger in men than in women [6]. However, studies investigating the relationship between living arrangement in later life and mortality have shown inconsistent results. Some authors report that living alone is associated with increased mortality, particularly in men [7–10] or in both genders with a stronger effect in men [11]. Other studies found no association between living alone and mortality [12, 13]. Men and women living alone in old age may be particularly more vulnerable due to poorer psychological health, functional limitations, lower economic, and social resources [14, 15]. Therefore, the effect of living alone on mortality in old age might possibly be amplified by the experience of feeling lonely. Despite a large number of studies addressing either living arrangment or loneliness on mortality risk, less is known about the combined effect of living alone and feeling lonely on risk for mortality. The present study aims to explore the role of living arrangement, as well as the combined condition of living arrangements and loneliness status as risk factors for all-cause mortality in a population-based cohort of septuagenarians. Further, we wanted to investigate whether results differ in men and women.

## Methods

### Study population

The Gothenburg H70 Birth Cohort Studies (the H70 studies) are ongoing population-based longitudinal studies of health and ageing. Full details of these studies have been reported elsewhere [16–20]. In brief, initiated first in 1971, the H70 studies are a series of epidemiological cohort studies of older men and women living in Gothenburg, Sweden. Seventy-year-old men and women listed in national population registers in Gothenburg were systematically selected based on specific birth dates. Participants underwent extensive medical, social, psychiatric, and physical examinations. The present study utilizes baseline data on 70-year-olds born in 1930 and interviewed in 2000 (*n* = 524, response rate 70%), as well as data on a 75-year-olds born in 1930 who were interviewed for the first time in 2005 (*n* = 329, response rate 68%). After excluding those with missing data on living alone and loneliness, a total of 778 participants remained for analysis (353 men, 425 women).

### Assessment of living alone and loneliness

Living alone was categorized as individuals who are single, or divorced, or widowed and live alone versus individuals who live with a partner (married/cohabiting) or with someone else.

Self-perceived feeling of loneliness was assessed by a single question as ‘do you feel lonely?’ There were four alternative responses, where 1 indicated never feeling lonely, 2 seldom, 3 sometimes, and 4 very often. The four categories were then merged into a dichotomous variable as 0 = not lonely (responses 1–2), and 1 = lonely (responses 3–4). This single item question to assess loneliness is the most common and widely used measure [21].

Further, men and women were categorized into the 4-groups depending on their living arrangement and loneliness status as follows: (1) living with someone and not feeling lonely, (2) living with someone and feeling lonely, (3) living alone and not feeling lonely, and (4) living alone and feeling lonely. These groups will be referred to as the 4-groups in the following sections.

### Mortality

Based on unique personal identification numbers and using the Swedish national population register, cohorts were followed for 6 years from the date of their baseline examination and until the end of the study.

### Other covariates

Current perceived economic situation was measured using seven response alternatives ranging from excellent to very bad (excellent, very good, good, average, not very good, bad, very bad). These responses were then merged into three categories and defined as: good (excellent, very good, good), average (average), and poor economic situation (not very good, bad, very bad). Smoking status was categorized as current smoker (regular or occasional), previous smoker, and never smoker. Physical activity was dichotomized into inactive (ie, no physical activity or sedentary most of the day) versus active (ie, regular nondemanding physically activities [eg, walks, gardening, dancing] 2–4 times per week, demanding physical activities [eg, tennis, running, swimming] at least 1 h/wk, or hard regular exercise). Alcohol consumption was measured with questions regarding weekly consumption of beer, wine, and spirits in centiliters (cl) during the past month. Based on these volumes, average weekly grams of alcohol consumption were calculated using conversion factors based on average alcohol concentration by volume (spirits 1 cl = 3 g, wine 1 cl = 1 g, beer > 3.5% 1 cl = 1/3 g). Heavy alcohol consumption for men and women aged 65 and over is defined as more than 98 g/wk by the National Institute on Alcohol Abuse and Alcoholism (NIAAA) (U.S. Department of Health and Human Services, National Institutes of Health, National Institute on Alcohol Abuse and Alcoholism, 2005). In this study, we categorized ≥ 100 g/wk alcohol consumption as risk consumption. Body mass index (BMI) was calculated from measured weight and height (weight in kg/height in m^2^), and obesity was defined as BMI ≥ 30 according to the criteria recommended by WHO [22]. Previous history of having (yes/no) cancer, diabetes, coronary heart disease, stroke, and chronic bronchitis was based on self-report as well as on medical examinations conducted by a study physician. Blood pressure was measured in the sitting position after a minimum of 5 min rest. Hypertension was defined by pharmacological treatment for hypertension or systolic blood pressure ≥ 140 mm Hg or diastolic blood pressure ≥ 90 mm Hg. Blood samples were drawn after an overnight fast from an antecubital vein and serum cholesterol measurements were determined according to standard laboratory procedures. Hypercholesterolemia was defined by pharmacological treatment and/or total fasting serum cholesterol ≥ 6.2 mmol/L. Impaired mobility was defined based on a six-item scale of activities of daily living (ADL) [23]. The ADL scale measured self-reported difficulties in performing daily life activities including transferring, dressing, bathing, using toilet, feeding, and continence [23]. Each item was coded as 0 = no need for help from another person, and 1 = need help. A composite index was created by summing up all the six items ranging from 0 to 6 (need no help to need help in all six activities). The index was then dichotomized as 0 (no impaired mobility) and 1 (impaired mobility, scale 1–6). Based on symptoms elucidated during a psychiatric examination, major depression was diagnosed according to the DSM-5 criteria (American Psychiatric Association, 2013) [24], and minor depression according to the DSM-IV-TR criteria (American Psychiatric Association, 2000) [25] using algorithms, as described previously [26]. Any depression was defined as presence of either minor or major depression. Definition of these depression variables has been described previously[27].

### Statistical analysis

**Fig. 1:**
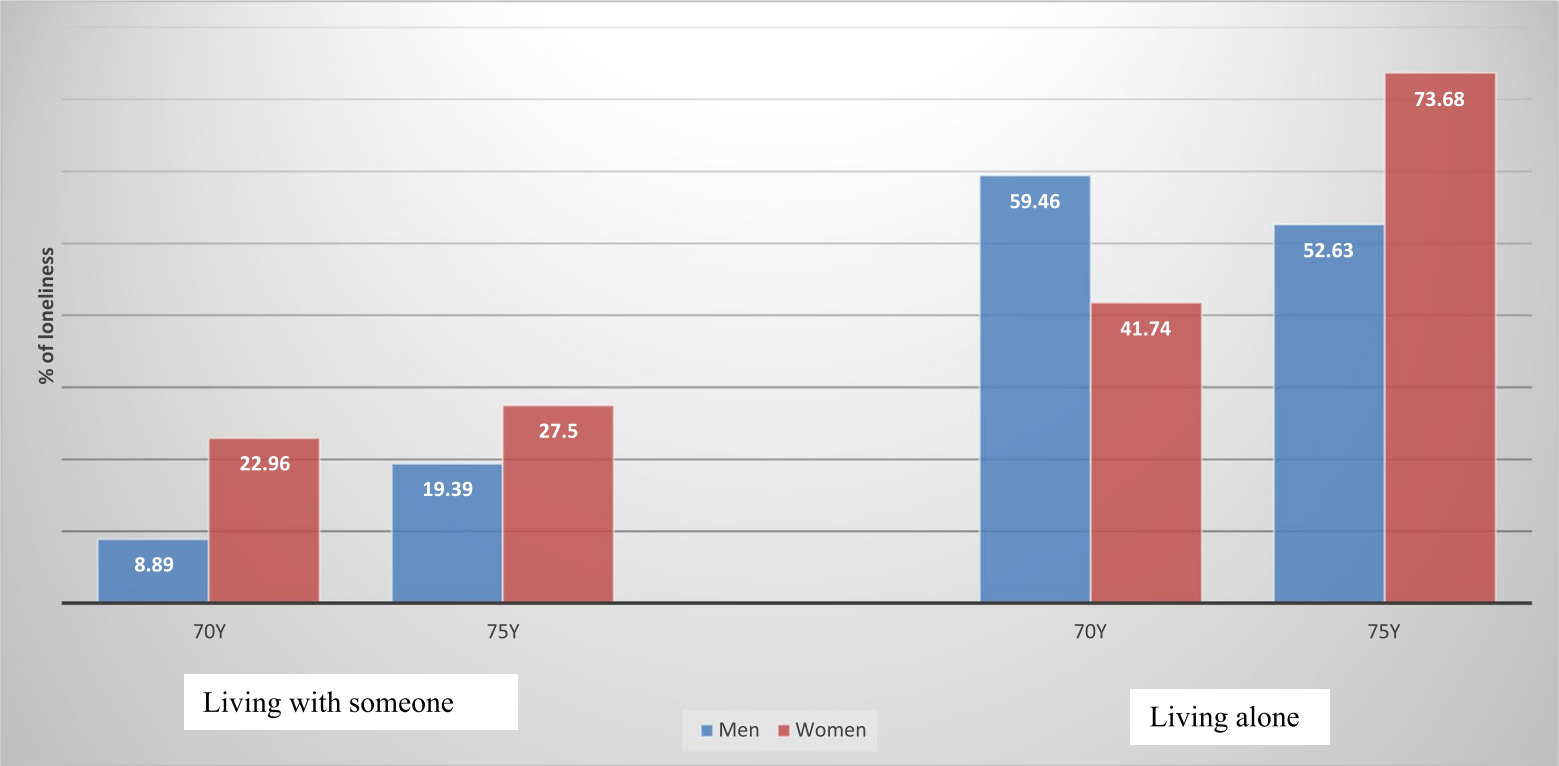
The proportion of men and women living with someone or alone and felling of loneliness according to age group

**Fig. 2:**
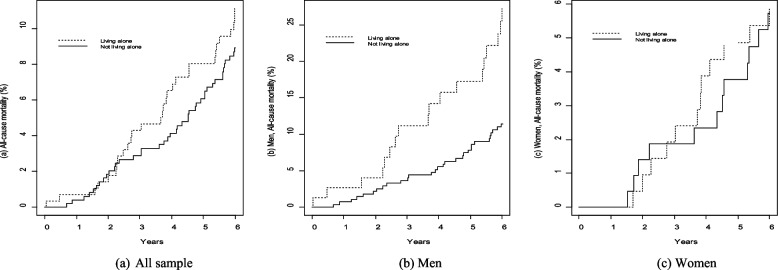
Kaplan–Meier curves for the cumulative incidence rates of all-cause mortality according to living arrangement, *P*-value = All sample 0.347, Men 0.0002, Women 0.948

**Fig. 3:**
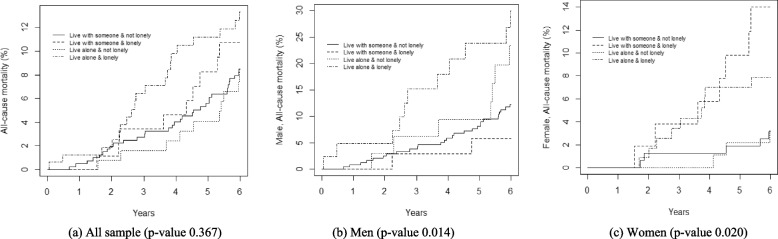
Kaplan–Meier curves for the cumulative incidence rates of all-cause mortality according to living arrangement and loneliness status

Statistical analyses were performed using SPSS, Windows version 25.0 (SPSS Inc., Chicago, IL, USA) and graphics were produced using R version 3.4.3 (The R Foundation for Statistical Computing). Due to smaller sample size, all the analyses were conducted by combining both age groups together, with the exception of baseline status of living arrangements and loneliness which are shown separately for the 70- and 75-year-olds in Fig. 1. The analyses were performed for men and women, for living alone and not living alone groups as well as for each of the 4-groups (living with someone and not lonely, living with someone and lonely, living alone and not lonely, and living alone and lonely) separately. We used three approaches for the data analysis. First, we assessed the distribution of the selected background factors between the groups (Table 1). The distributions of the factors were expressed in percentages and the differences between groups were tested using Pearson × 2-tests. A *p-value* of < 0.05 was considered significant. Secondly, the survival functions for the 6-year period were assessed using the Kaplan Meier method, and log-rank tests were used to evaluate the group differences (Figs. 2 and 3). In the last step, Cox proportional hazard regression models were used to study the associations between living alone and 6-year mortality (Table 2), as well as between the 4-groups and 6-year mortality (Table 3). Both unadjusted and multivariable adjusted regressions were carried out. Factors that were shown to be associated with living alone as well with the 4-groups (from Table 1) were included in multivariable models. Estimates derived from Cox regressions are presented as hazard ratios (HR) and 95% confidence intervals (CI).

**Table 1:** Distribution of baseline characteristics among men according to their living arrangement and perceived loneliness status

Men	Total *N* = 353	Living with someone			Living alone			*P-value*	
		Not lonely *N* = 243	Lonely *N* = 35	All *N* = 278	Not lonely *N* = 33	Lonely *N* = 42	All *N* = 75	Living with someone vs. living alone	Between the 4-groupsª
Perceived poor economic situation	9.2 (32)	6.7 (16)	11.4 (4)	7.3 (20)	9.4 (3)	22.0 (9)***	16.4 (12)	0.005	0.006
Loneliness	21.8 (77)	--	--	12.6 (35)	--	--	56.0 (42)	0.000	--
Cardiovascular risk factors:									
Hypertension	84.8 (289)	86.1 (205)	88.2 (30)	86.4 (235)	83.3 (25)	74.4 (29)	79.3 (54)	0.071	0.264
Hypercholesterolemia	34.3 (121)	38.3 (93)	31.4 (11)	37.4 (104)	36.4 (12)	11.9 (5)***	22.7 (17)	0.017	0.010
Current smoker	12.3 (42)	11.3 (27)	14.7 (5)	11.8 (32)	13.3 (4)	15.4 (6)	14.5 (10)	0.449	0.877
Physically inactive	8.7 (29)	8.5 (20)	12.1 (4)	9.0 (24)	3.4 (1)	10.5 (4)	7.5 (5)	0.692	0.645
Alcohol consumption ≥100 gm/wk	25.2 (84)	26.0 (60)	12.1 (4)	24.2 (64)	30.0 (9)	28.2 (11)	29.0 (20)	0.438	0.306
Obese. BMI ≥30	17.4 (61)	17.5 (42)	8.6 (3)	16.4 (45)	24.2 (8)	19.0 (8)	21.3 (16)	0.457	0.459
Diabetes	12.9 (44)	12.2 (29)	8.8 (3)	11.8 (32)	13.3 (4)	20.5 (8)	17.3 (12)	0.213	0.453
CHD	25.7 (88)	25.1 (60)	14.7 (5)	23.8 (65)	29.0 (9)	35.9 (14)	32.9 (23)	0.122	0.212
Stroke	6.2 (21)	5.9 (14)	11.8 (4)	6.6 (18)	6.7 (2)	2.6 (1)	4.4 (3)	0.499	0.440
Chronic physical and mental health condition:									
Cancer	17.6 (60)	18.1 (43)	20.6 (7)	18.4 (50)	10.3 (3)	17.9 (7)	14.5 (10)	0.449	0.695
Chronic bronchitis	14.7 (50)	15.1 (36)	11.8 (4)	14.7 (40)	13.3 (4)	15.4 (6)	14.5 (10)	0.964	0.955
Impaired mobility	10.3 (34)	9.2 (21)	3.1 (1)	8.5 (22)	12.9 (4)	20.5 (8)*	17.1 (12)	0.034	0.082
Depression	11.8 (41)	5.0 (12)	40.0 (14)***	9.5 (26)	15.6 (5)**	23.8 (10)***	23.8 (15)	0.011	0.000
Women	Total *N* = 425	Living with someone			Living alone			*P-value*	
		Not lonely *N* = 162	Lonely *N* = 53	All *N* = 215	Not lonely *N* = 92	Lonely *N* = 118	All *N* = 210	Living with someone vs. living alone	Between the 4-groupsª
Perceived poor economic situation	10.9(46)	3.7 (6)	9.4 (5)**	5.1 (11)	15.2 (14)***	17.9 (21)***	16.7 (35)	0.000	0.000
Loneliness	40.2 (171)	--	--	24.7 (53)	--	--	56.2 (118)	0.000	--
Cardiovascular risk factors:									
Hypertension	80.5 (321)	81.9 (127)	78.4 (40)	81.1 (167)	83.7 (72)	76.6 (82)	79.8 (154)	0.423	0.584
Hypercholesterolemia	53.9 (229)	54.9 (89)	50.9 (27)	54.0 (116)	60.9 (56)	48.3 (57)	53.8 (113)	0.976	0.315
Current smoker	13.0 (52)	5.8 (9)	21.6 (11)***	9.7 (20)	17.4 (15)**	15.7 (17)***	16.5 (32)	0.015	0.001
Physically inactive	9.9 (39)	6.5 (10)	22.0 (11)***	10.3 (21)	4.7 (4)	13.2 (14)	9.4 (18)	0.772	0.003
Alcohol consumption ≥100 gm/wk	10.5 (42)	12.4 (19)	8.3 (4)	11.4 (23)	7.0 (6)	11.5 (13)	9.5 (19)	0.625	0.553
Obese. BMI ≥30	20.1 (85)	18.5 (30)	17.3(9)	18.2 (39)	24.2 (22)	20.5 (24)	22.1 (46)	0.794	0.349
Diabetes	9.5(38)	7.1 (11)	17.6(9)*	9.8 (20)	11.6 (10)	7.4 (8)	9.3 (18)	0.871	0.115
CHD	19.4(78)	15.5 (24)	17.3 (9)	15.9 (33)	24.4 (21)	22.0 (24)	23.1 (45)	0.071	0.317
Stroke	5.3 (21)	5.2 (8)	7.8 (4)	5.8 (12)	4.7 (4)	4.7 (5)	4.7 (9)	0.603	0.844
Chronic physical and mental health condition:									
Cancer	16.3 (65)	15.6 (24)	29.4(15)*	9.8 (39)	12.8(11)	13.9 (15)	6.5 (26)	0.129	0.052
Chronic bronchitis	17.3 (69)	7.7 (12)	29.4 (15)***	13.1 (27)	22.1 (19)***	21.3 (23)***	21.6 (42)	0.024	0.000
Impaired mobility	11.5 (46)	10.1(15)	13.7 (7)	11.0 (22)	4.0 (8)	14.5 (16)	12.1 (24)	0.740	0.552
Depression	20.2(85)	6.2 (10)	37.7 (20)***	14.0 (30)	13.5(12)*	36.4 (43)***	26.6 (55)	0.001	0.000

Values presented in this table are percentage with number of subjects in parenthesis. *BMI* body mass index (weight in kg/height in m²). *CHD* coronary heart disease. ªThe 4-groups: Living with someone and not lonely (group 1), living with someone and lonely (group 2), living alone and not lonely (group 3), living alone and lonely (group 4)

*, **, *** = *p*-value 0.05, *p*-value 0.01, *p*-value 0.001, and the comparisons are made with group 1, values without these signs are not significant

**Table 2:** Cox proportional hazard ratios (HR) with 95% confidence intervals (CI) of 6-years mortality in relation to living arrangement

	**Living arrangement **	
	**Live with someone**	**Live alone**
**Men**		
Unadjusted	1.0	2.40 (1.34-4.30)
Basic model (age adjusted)	1.0	2.13 (1.18-3.85)
Basic model plus:		
Poor socioeconomic situation	1.0	2.20 (1.19-4.02)
Loneliness	1.0	2.24 (1.18-4.27)
Hypercholesterolemia	1.0	2.11 (1.16-3.84)
Impaired mobility	1,0	2.25 (1.21-4.21)
Depression	1.0	2.07 (1.14-3.77)
All factors	1.0	2.56 (1.27-5.16)
**Women**		
Unadjusted	1.0	1.03 (0.46-2.29)
Basic model (age adjusted)	1.0	0.93 (0.42-2.08)
Basic model plus:		
Poor socioeconomic situation	1.0	0.88 (0.38-2.06)
Loneliness	1.0	0.63 (0.27-1.49)
Smoking	1.0	1.05 (0.43-2.55)
Chronic bronchitis	1.0	1.05 (0.43-2.57)
Depression	1.0	0.86 (0.38-1.93)
All factors	1.0	0.73 (0.28-1.90)

Only significant variables from Table 1 are selected for adjustments

**Table 3:** Cox proportional hazard ratios (HR) with 95% confidence intervals (CI) of 6-years mortality in relation to living arrangement and perceived loneliness status

	**Living arrangement and loneliness**			
	**Live with someone and not lonely**	**Live with someone and lonely**	**Live alone and not lonely**	**Live alone and lonely**
**Men:**				
Unadjusted	1.0	0.48 (0.11-2.03)	1.90 (0.83-4.35)	2.52 (1.26-5.07)
Basic model (age adjusted)	1.0	0.41 (0.10-1.73)	1.62 (0.70-3.75)	2.18 (1.08-4.43)
Basic model plus:				
Poor socioeconomic situation	1.0	0.41 (0.10-1.74)	1.66 (0.72-3.84)	2.29 (1.10-4.76)
Hypercholesterolemia	1.0	0.41 (0.10-1.73)	1.61 (0.70-3.74)	2.16 (1.05-4.44)
Depression	1.0	0.32 (0.07-1.42)	1.56 (0.67-3.63)	1.94 (0.94-4.01)
All factors	1.0	0.32 (0.07-1.40)	1.59 (0.68-3.71)	2.04 (0.95-4.37)
**Women:**				
Unadjusted	1.0	4.52 (1.43-14.23)	1.05 (0.25-4.40)	2.55 (0.86-7.63)
Basic model (age adjusted)	1.0	4.15 (1.32-13.09)	1.16 (0.28-4.88)	1.99 (0.66-6.01)
Basic model plus:	1.0			
Poor socioeconomic situation	1.0	4.11 (1.29-13.12)	1.15 (0.27-4.91)	1.97 (0.62-6.25)
Smoking	1.0	5.07 (1.25-20.46)	1.75 (0.35-8.61)	2.64 (0.69-10.20)
Physical inactivity	1.0	3.22 (0.73-14.31)	2.10 (0.42-10.42)	2.42 (0.62-9.48)
Cancer	1.0	4.98 (1.23-20.09)	1.96 (0.40-9.74)	3.32 (0.87-12.71)
Chronic bronchitis	1.0	4.66 (1.12-19.43)	1.74 (0.35-8.77)	2.63 (0.68-10.29)
Depression	1.0	3.69 (1.11-12.23)	1.15 (0.27-4.83)	1.80 (0.57-5.65)
All factors	1.0	1.90 (0.40-9.16)	1.43 (0.27-7.69)	2.03 (0.47-8.82)

Only significant variables from Table 1 are selected for adjustments

## Results

Of the 778 participants, 467 were 70-year-olds (60%) and 425 were women (54.6%). Just over a third (*n* = 28,536.6%) lived alone, with a higher prevalence in women than in men (49.4% versus 21.2%; *p* = 0.000). The prevalence of living alone was 32.5% at age 70 (men 17.1%, women 46.0%) and 42.8% at age 75 (men 27.9%, women 54.3%), respectively.

At baseline, 31.9% reported feelings of loneliness (women 40.2%, men 21.8%, *p* = 0.000). The association between living arrangement and feeling of loneliness is displayed in Fig. 1 by age group. Among 70-year-olds who lived with someone, 23% of women and 8.9% of men reported feeling lonely (*p* = 0.001). Among those who lived alone, 41.7% of women and 59.6% of men reported feeling lonely (*p* = 0.060). Among 75-years-olds who lived with someone, the prevalence of loneliness did not differ between men and women (19.4% versus 27.5%, *p* = *0.201*). However, among those who lived alone 52.6% of men and 73.7% of women reported feeling lonely (*p* = *0.019*).

Among men, living alone was associated with perceived poor economic situation, impaired mobility and depression. Further, men who lived alone were less likely to have hypercholesterolemia compared to men living with someone (Table 1). Women living alone more often perceived their economic situation as poor, were smokers and had chronic bronchitis, and depression compared to women living with someone.

Both in men and women, no significant differences were observed between those who lived alone or with someone regarding hypertension, physical inactivity, risk consumption of alcohol, obesity, diabetes, CHD, stroke, and cancer. Additionally, for women, there were no differences regarding hypercholesterolemia and impaired mobility between those living alone and those living with someone. Furthermore, for men, there were no differences regarding chronic bronchitis and smoking between those living alone and those living with someone.

Both in men and women, most of the health-related factors did not differ between the 4-groups (Table 1). In men, significant differences were observed only in poor perceived economic situation, hypercholesterolemia, and depression, but the associations were not consistent. For instance, poor economy was most common among the group of men living alone and feeling lonely followed by the group living with someone and feeling lonely, and the lowest was among the group living with someone and not feeling lonely. Hypercholesterolemia on other hand was most common among men living with someone and not feeling lonely followed by living alone and not feeling lonely and the lowest was among men living alone and feeling lonely. Depression (major or minor) was found to be most prevalent in men living with someone but feeling lonely followed by living alone and feeling lonely and the lowest prevalence was among men living with someone and not feeling lonely.

In women, consistently highest prevalence for smoking, physical inactivity, cancer, chronic bronchitis, and depression were found among those living with someone and feeling lonely compared to the other three groups (Table 1). Poor economic situation was most common among women living alone and feeling lonely followed by living alone and not feeling lonely, and the lowest was among women living with someone and not feeling lonely.

### Total mortality in men and women:

A total of 72 (9.3%) participants died during the 6-year follow-up period, with a median follow-up of 5.7 years and 4 506 person-years at risk, corresponding to 16.0 deaths per 1000 person-years. The cumulative mortality rates were 23.9/1000 person-years for men and 9.6/1000 person-years for women.

### Mortality in men and women living with someone versus living alone

Kaplan Meier analyses showed no significant differences between the groups when men and women were analyzed together (Fig. 2a). However, stratified analyses showed a lower survival rate among men who lived alone compared to those who lived with someone (*p* = *0.002*) (Fig. 2b), while no such difference was observed in women (*p* = *0.948*) (Fig. 2c).

Similar to Kaplan Meier results, Cox regression analyses showed that men who lived alone had a more than twofold risk of mortality compared to those who lived with someone (HR 2.40, 95% CI 1.34–4.30), and the risk remained significant in a multivariable-adjusted model (HR 2.56, 95% CI 1.27 – 5.16). No significant increased risk of mortality was observed when women who lived alone were compared to those who lived with someone (HR 1.03 95% CI 0.46–2.29) (Table 2).

Kaplan Meier analysis showed lowest survival rate among men who lived alone and felt lonely (*p* = *0.014*) (Fig. 3b). Among women, the lowest survival rate was observed among those who lived with someone, but felt lonely (*p* = *0.020*) (Fig. 3c).

In Cox regression analyses, men who lived alone and felt lonely had a more than two-fold risk of mortality compared to men who lived with someone and did not feel lonely (HR 2.52, 95% CI 1.26 – 5.05) (Table 3). The risk of mortality was more than four times higher among women who lived with someone but felt lonely, compared to those who lived with someone and did not feel lonely (HR 4.52, 95% CI 1.43 – 14.23). In the multivariable adjusted models these associations were mainly attenuated by depression in men and by physical inactivity in women.

## Discussion

In this Swedish population-based study of septuagenarians, one fourth of the women reported feeling lonely despite living with someone. This group had the highest mortality, a rate more than four times that compared to women living with someone and not feeling lonely. In contrast, among men, those living alone and feeling lonely had the highest mortality. When considering only living arrangement, we found that living alone was an independent risk factor for death in men, but not in women.

The present study reflects the worldwide observation that living alone is more common among women than among men aged over 65, which is one of the most visible characteristics of societal aging [28]. Our study confirms previous findings of an excess mortality in men who lived alone even after controlling for a variety of confounders, including loneliness and depression [7–10]. The explanation for the increased mortality in men living alone is likely multifactorial. The presence of a partner can foster healthy behaviors such as not smoking, regular exercise and balanced diet [29–31], can facilitate seeking emergency care in case of accidents or acute events, such stroke or myocardial infarction [32], facilitate early diagnosis of various medical conditions, such as cancer or dementia [33], and give better adherence to treatment for medical conditions [34].

As both loneliness [6] and living alone have been shown to be associated with mortality [7–11, 35, 36], our assumption was that older adults living alone and feeling lonely are worse off compared to those who live with someone. This assumption was true only for men in our sample. Among women, however, those who lived with someone but felt lonely had the highest risk of death. In general, previous studies tend to focus on either loneliness or living alone, while studying mortality risk. To date, very few studies have investigated the risk of mortality for both genders separate in relation to the combination of living arrangement and loneliness status, making it difficult to compare our study results to those of others. One Chinese study found that people who lived with others but felt lonely had higher mortality [36] Another German study found that subjective loneliness is associated with an increased risk of all-cause mortality in older adults who lived alone[37]. Our study results show the importance of considering both loneliness and living alone in assessing mortality risk for men women in old age.

The mechanism by which women living with others but feeling lonely had increased risk of death in our study is not clear. One possible explanation could be that this group had worse health status and health related behavior compared to the other three groups, e.g. regarding smoking, physical inactivity, diabetes, cancer, chronic bronchitis, and depression. When controlling for all these factors, the associations were attenuated and were no longer significant. The most important factor was physical inactivity, which was more common in this group of women. The presence of poorer health may have led to lack of physical activity. The higher prevalence of loneliness among these women could also be related to being caregiver for a sick partner. Having poor health or being a caregiver for an ill spouse or other kin could restrict physical activity and social contacts, hence increase the risk of loneliness, which in turn can negatively influence survival in old age.

The strengths of the study included the population-based samples of both men and women and the comprehensive examinations with data on socio-demographic factors, medical history, and clinical- and physical measurements. However, this study also had a number of limitations. First, although the response rate at baseline is higher (70% at age 70, and 68% at age 75) than in many other studies [38], we cannot exclude the possibility that participants were healthier than non-participants which might have caused selection bias. Second, loneliness was assessed with a single-item direct question. This may result in underreporting due to the stigma associated with being identified as lonely [39–41], particularly among men [41]. It has been shown that loneliness is more prevalent among men when using the indirect measure [41]. Whereas using a direct measure, loneliness is more prevalent among women [41]. This may imply that lonely men are underrepresented in our sample as compared to women. Furthermore, implying that the direct measurement used in our study may not have captured the overall influence of loneliness on mortality in men. This single item direct question of loneliness, however, is the most common and widely used measure [21], which previously has been shown to predict mortality [42–45]. Another limitation was the use of a one-time assessment of living arrangement and loneliness status at baseline. As both loneliness and living arrangements are dynamic over time, changes in living arrangements or loneliness status during the six years of follow-up could not be taken into consideration in our study. As men and women respond differentially depending on methods used to assess loneliness, in the future research, more dimensions of loneliness as well as dynamic changes of loneliness and living arrangements should be further investigated separately for men and women.

In conclusion, living alone was an independent risk factor for death in men but not in women. Mortality risk was doubled in men who lived alone and reported loneliness. For women, mortality was particularly elevated in those who lived with others and felt lonely. These associations were attenuated and were no longer significant after adjusting for mainly depression in men and physical inactivity in women. Gender needs to be taken into account when considering the health consequences of living situation and loneliness.

## Data Availability

The data supporting this article can be made available from the corresponding author on reasonable request.
